# 'Team capital’ in quality improvement teams: findings from an ethnographic study of front-line quality improvement in the NHS

**DOI:** 10.1136/bmjoq-2020-000948

**Published:** 2020-05-26

**Authors:** Catherine Montgomery, Stephen Parkin, Alison Chisholm, Louise Locock

**Affiliations:** 1 Centre for Biomedicine, Self and Society, University of Edinburgh, Edinburgh, UK; 2 National Addiction Centre, Institute of Psychiatry, Psychology & Neuroscience, King's College London, London, UK; 3 Nuffield Department of Primary Care Health Sciences, University of Oxford, Oxford, UK; 4 Health Services Research Unit, University of Aberdeen, Aberdeen, UK

**Keywords:** quality improvement, patient-centred care, teamwork, qualitative research

## Abstract

**Background:**

Teamwork is important in the design and delivery of initiatives in complex healthcare systems but the specifics of quality improvement (QI) teams are not well studied.

**Objective:**

To explain the functioning of front-line healthcare teams working on patient-centred QI using Bourdieu’s sociological construct of capital.

**Methods:**

One medical ward from each of six NHS Trusts in England participated in the study, purposively selected for a range of performance levels on patient experience metrics. Three ethnographers conducted focused ethnography for 1 year, using interviews and observations to explore the organisation, management and delivery of patient-centred QI projects by the six front-line teams. Data were analysed using Bourdieu’s typology of the four forms of capital: economic, social, symbolic and cultural.

**Results:**

While all teams implemented some QI activities to improve patient experience, progress was greater where teams included staff from a broad range of disciplines and levels of seniority. Teams containing both clinical and non-clinical staff, including staff on lower grades such as healthcare assistants and clerks, engaged more confidently with patient experience data than unidisciplinary teams, and implemented a more ambitious set of projects. We explain these findings in terms of ‘team capital’.

**Conclusion:**

Teams that chose to restrict membership to particular disciplines appeared to limit their capital, whereas more varied teams were able to draw on multiple resources, skills, networks and alliances to overcome challenges. Staff of varying levels of seniority also shared and valued a broader range of insights into patient experience, including informal knowledge from daily practice. The construct of ‘team capital’ has the potential to enrich understanding of the mechanism of teamwork in QI work.

## Introduction

Much quality improvement (QI) work in healthcare is focused on improving clinical outcomes and patient safety, and making efficient use of resources. Improving patient experience of care has received less attention, but is increasingly recognised as an important component of quality. Front-line staff charged with improving patient experience may have little experience or expertise in QI, and the kinds of teamwork practices required to achieve it. While there is substantial literature around effective teamwork in healthcare generally,^1–10^ the specifics of QI teams are less well studied. Staff who may be well used to teamworking in the context of clinical care may find these patterns of working relationships do not transfer straightforwardly to QI, where the issues may be different. A recent scoping review on dynamics in QI teams^11^ notes that:

These teams are often ad hoc collections of various professions and/or occupations, working together in time-limited ways to accomplish specific QI aims. Much of the QI enterprise relies on the ability of these teams to identify a problem, design a solution, lead tests of change and implement a sustainable quality plan. Despite their pivotal role, little scholarly attention is paid to the processes of these teams.

As Rowland *et al*^11^ point out, ‘a functional QI team alone is unlikely to create change’. They argue that the impact of both interprofessional representation on QI teams and organisational contexts require further research.

In this paper, we report findings from research into how front-line NHS staff use patient experience data to improve care. Our analysis responds directly to Rowland *et al*’s call above by introducing the concept of ‘team capital’ to explain how teams effect change in QI projects. While we have referred to this concept elsewhere,^12^ in this paper, we develop the theoretical basis of our findings, and illustrate this with empirical examples.

The term ‘team capital’ draws on Bourdieu’s work describing how social relationships are shaped and organised by access to assorted resources.^13 14^ For Bourdieu, capital is the key driving mechanism for establishing, maintaining and reproducing social relationships within and across different fields of practice. In addition, capital provides the bases for advancing individual position and status within and across these relationships, in which the reproduction of capital typically serves to increase social standing within a given milieu (whether social class, professional network or peer group). Bourdieu asserts that habitus (conscious and unconscious behaviours associated with particular sociocultural settings), field (values, rules and norms associated with symbolic systems) and capital are conjoined ‘structuring structures’ that are inextricably linked to one another in symbiotic and symbolic relationships that are able to surpass multiple fields.

Within Bourdieu’s schema, capital exists in three distinct ‘states’: an objectified state (visible, material assets), an embodied state (unconscious and conscious knowledge associated with habitus and techniques of the body) and an institutionalised state (knowledge, skills and behaviours associated with personal/professional identity). Finally, Bourdieu outlines a typology of four *forms* of capital that exist within each of these *states*, namely social, symbolic, cultural and economic capital.

### Habitus, field and capital in the NHS

As with most institutions, the English NHS is organised by a hierarchical staff grading system. In 2017, when this research was conducted, this scale consisted of nine Band levels, containing a total of 54 specific points that applied to all divisions and sectors (clinical and non-clinical) throughout the service. The 2017 NHS entry level salary was £15 404 (Band 1 Point 2) compared with the highest point at £100 431 (Band 9 Point 54).^15^ The banding system within the NHS reflects a ranked structure in work-related roles and associated pay scales, which in turn establishes a system for the structural organisation of clinical and non-clinical positions throughout the health service. These features of a nationwide organisational process automatically establish habitus (unconscious and conscious behaviours associated with individual roles/positions), field (the values, norms and expectations of NHS membership—as outlined in the NHS Constitution) and practice (or ‘professional culture’). The NHS band system and Bourdieu’s construction of capital are therefore pertinent when considering the functioning of teams on the NHS front line.

## Methods

In 2015, The NHS National Institute for Health Research commissioned a suite of studies about how best to use patient experience data for QI, including the study reported here. The funding call reflected the concern that patient experience is a neglected aspect of quality compared with effectiveness and safety. Our study focused on whether/how front-line staff in hospital general medical wards used patient experience data, with comparative case studies in six acute hospitals in England. For a full description of the methods, please see Locock *et al*.^12 16^

Following an invitation to participate in the study, the selected NHS Trusts each identified a medical ward suitable for addressing the research question of how front-line staff use patient experience data for QI. Each ward assembled a core team of up to five members responsible for the design, delivery and implementation of patient experience improvement projects. The teams were given information on different types of patient experience data (such as survey findings, patient stories, Friends and Family Test and ward observations) and how these can be used for QI at a 2-day learning event. They were then asked to design and implement their own QI activities on returning to their hospital wards. A team of three ethnographers, with PhDs in sociology and psychology, and diverse previous health research experience, observed this process over the course of a year (July 2016–September 2017), adopting a focused ethnography approach.^17–19^ This involved observing local QI meetings, meetings of patient and carer experience groups, general staff meetings and workspaces and chatting informally with staff. Ninty-five semistructured audiorecorded interviews were conducted at the start, midpoint and end of fieldwork (Table 1), covering topics such as staff’s experiences of conducting QI work, factors affecting the project (both positively and negatively) and perceived impacts of working together as a team to improve care based on patient experience data. All participants were sent an information sheet explaining the study before consenting to take part.

**Table 1:** Data collection

	Core team interview baseline	Core team interview midpoint	Core team interview end of study	Senior/other interviews	Total interviews	Number of site visits	Total hours of observation
Site 1	4	0	4	10	18	7	45
Site 2	5	0	3	5	13	8	48
Site 3	6	2	5	5	18	8	54
Site 4	7	5	3	3	18	12	58
Site 5	5	2	2	2	11	8	48
Site 6	5	2	4	6	17	8	46
Total	32	11	21	31	95	51	299

The ethnography was designed to be formative, with two further learning community events at mid and endpoint. At the midpoint event, the research team shared emerging reflections with team members and invited discussion of challenges encountered and how these might be addressed.

To enable participants to share potentially negative or critical views, sites have not been named or identified and all respondents are referred to in terms of their (preferred and self-described) professional position.

### Data analysis

All fieldnotes and interviews were transcribed, pseudonymised and entered into NVivo V.10/11 for analysis, concurrent with ongoing fieldwork. The ethnography team held regular ‘data sessions’ to share reflections^18^ and a common coding framework was inductively generated. This included types of data used; attitudes towards/understanding of data; team composition and membership; relationships with patient experience office and senior management; and organisational pressures and constraints. The coding framework allowed for nuanced coding of organisational culture and practices, including hierarchies and power relationships, time and priorities and the composition/allocation of roles. Following thematic coding by each ethnographer of interviews and ethnographic fieldnotes, detailed descriptions of events, timelines, actors and context in each case study site were developed to enable overarching cross-case comparison and a focus on the process of change as advocated by Pettigrew *et al*.^20^ These formed the basis of iterative data analysis discussions, during which team composition was identified as a key factor which helped explain what happened in the different sites. As a way to make sense of this finding, we subsequently applied the sociological construct of capital to our analysis: the extent to which a team commands varied practical, organisational and social resources which enable them to set agendas, drive processes and implement change. These include not just material or economic resources, but also status, time, space, relational networks and influence.

### Theoretical framework: Bourdieu’s ‘capital’

As outlined above, Bourdieu’s^13 14^ concept of capital focuses on how social relationships are shaped and organised by access to resources (broadly understood) within and across different fields of practice. Economic capital relates to physical objects and artefacts that demonstrate some form of visible ‘wealth’ within a particular field of practice. Social capital relates to the range of social networks and alliances available at an individual or organisational level. It concerns relational and embodied characteristics of agency and may include qualities such as leadership, empathy, reciprocity and trust. Symbolic capital relates to an individual’s reputation, prestige, honour and status within a social setting. Cultural capital is typically made manifest in the overt/covert demonstration of expertise in, or knowledge of, particular forms of practice within a given milieu.

In applying a Bourdieusian lens to our data, we sought to explain the varying levels of progress between teams as they designed and implemented patient experience-based QI in front-line settings (see table 2). In the process, the salience of these sociological concepts became clear; for example, we noted how the expertise and knowledge conferred by cultural capital related to the clinical and medical procedures associated with particular wards or patient groups. Similarly, non-clinical expertise in QI methods, such as generating, analysing and disseminating patient experience data, reflected another form of cultural capital relevant to other sectors throughout the NHS. We designated as ‘team capital’ the assorted resources, skills, knowledge and experience, united as one, within a multidisciplinary team of professionals from clinical and non-clinical backgrounds and from a variety of Band levels. In adding the prefix ‘team’, we foreground the crucial unit of the team in healthcare settings and hope thereby to enable greater engagement between health services research and Bourdieusian insights.

**Table 2:** Team composition, capital and improvements made

Team	Team composition	Generation of team capital	Improvements made
1	Unidisciplinary, low grades: three healthcare assistants and two nurses	Low	One intervention: introduction of a welcome pack for patients.
2	Multidisciplinary, mixed grades: ward nursing manager, ward clerk, healthcare assistant and patient experience officer	High	Various and wide-ranging incl: admission packs; staff training; information boards and leaflets; interventions to encourage communication between patients and staff, bedside whiteboards and a ‘what matters to me’ board for patient and staff comments.
3	Multidisciplinary, mixed grades: ward nursing manager, activities and well-being coordinator, head of QI and patient experience and a patient experience officer	High	Multiple interventions incl: design of a welcome pack for new patients and a discharge pack; a photo board to help patients/relatives identify staff (and their role); call bell use and response times; socialised dining in the ‘social room’; introduction of a structured activities timetable and bespoke activities; increased 1–1 time with the activities and well-being coordinator.
4	Multidisciplinary, mixed grades: ward manager, nursing staff, senior healthcare assistant, patient experience project manager. Later, a pharmacist, medical registrar, charge nurse and junior doctors	High	Ambitious redesign of discharge process, including prescribing, to be more efficient following a series of successful stakeholder mapping sessions; design of information leaflets for patients covering the ward’s most frequent conditions.
5	Unidiscplinary, mixed grades: ward manager, two nurses and two healthcare assistants	Medium	Various small-scale changes incl: sympathy cards for bereaved relatives; promoting (and auditing the use of) sleep-well packs to address noise at night; having nurses accompany doctors on ward rounds to help with patient understanding and communication; mainstreaming patient experience into routine ward practices.
6	Unidiscplinary, high grades: two quality nurses, matron, ward nursing manager and associate chief nurse for medicine	Low	Small-scale changes, incl: reorganisation of communication at shift handover; ward-based ‘It’s OK to Ask’ scheme (to improve patient–staff communication); trial run of ‘sleep packs’ for patients. Many of the team’s original plans were not delivered on.

QI, quality improvement.

### Patient and public involvement

The research team was advised by a lay panel of 10 people, chaired by the project’s lay coinvestigator, who was involved from the start, contributing to the study design, drafting the funding proposal and reviewing the protocol. Lay panel members all had recent personal or family experience of inpatient care. They attended all three learning communities, and met regularly with the principal investigator and other members of the research team to reflect on and make sense of emerging findings.

## Results

It was anticipated that some teams would make greater progress than others, given our varied purposive sampling strategy. While all teams implemented some QI activities to improve patient experience, team composition seemed to be more important in explaining the results than prior performance or recent organisational history. For example, two sites which were both emerging from recent challenging inspections and poor performance performed radically differently. As membership of each team was determined locally, there was variation in professional role, discipline, level of seniority and clinical/non-clinical experience across the six teams. Two unidisciplinary teams consisted almost entirely of nursing staff from similar relatively senior grades, in contrast to four teams comprising clinical and non-clinical staff (from unqualified healthcare assistants and ward clerks through to senior managers and consultants). Some included members from the hospital’s central patient experience function in the core QI team. Despite encouragement to involve patients as core team members, direct involvement of patients and relatives was minimal or non-existent.

In terms of the number and scope of improvements made, progress was generally greater where teams included a broad range of clinical and non-clinical staff from multiple disciplines and levels of seniority, and when the team included patient experience office staff (see table 2). (All the case study sites in our sample had a designated patient experience office, though not always with a remit for QI). We summarise below how the range of different forms of capital associated with each ward team contributed to the progress of the various improvement projects. In each instance we examine positive and negative capital. Although we discuss the forms of capital separately, in practice they were often overlapping and mutually reinforcing.

### Access to resources—the role of economic capital

Economic capital denotes the assets and resources teams could command, such as infrastructure (including office space), equipment and materials and staff capacity and time. The adverse impact of severe winter pressures during the study reduced the capacity and availability of some clinical staff to fully engage with QI. In one site, ward-based resources (office space and equipment) were made available to a staff member from the patient experience office, and his presence as an extra, non-clinical person on the ward enabled QI work to continue while clinical staff managed increased demands on their time. The ward manager’s economic capital (in the form of office space) facilitated work by the patient experience officer (who brought economic capital in the form of time) for the benefit of the whole team.

As a result of the convergence of economic capital held by individuals from different departments, the collective capital of the front-line team was amplified, which in turn facilitated continued participation in the field of practice (the development of QI projects within front-line settings). The active involvement of patient experience staff did not just bring economic capital in the form of staff time, but also other forms of social and cultural capital discussed below.

Conversely, several sites reported difficulties accessing key resources, including small amounts of money to pay for equipment or photocopying, lack of physical space to meet and lack of staff time. Reduced access to such assets may disable participation in improvement work. For example, one healthcare assistant explained how they were unable to access work-IT from home, unlike some more senior nursing staff. The lack of privileges associated with computer access (reduced symbolic capital within the organisation) prevented communication with higher-level colleagues when off-site, hampering the team’s collective ability to make progress. In another site, the teams were observed to meet off-site in the evenings, due to a lack of office space and reduced staff capacity during the working day.

Resource pressures at ward level are a long-standing problem in the NHS and affected all the teams to some degree. However, teams with more diverse membership were sometimes able to draw on other forms of capital to access resources. For example an activities coordinator and a ward clerk (both relatively junior in the organisation) were able to bring time and creative materials to the collective QI work of the teams. More unidisciplinary teams were reliant on a narrower range of avenues to garner economic capital.

### Networks and relationships—deploying social capital

Social capital relates to the range of social networks and alliances available at an individual or organisational level. Through social capital, individuals can exploit and maximise their own position within a given network as well as accrue further social capital for collective team benefit. For example, the head of patient experience in one site explained how distributed leadership between herself, the ward manager and a patient experience officer jointly secured access to networks and resources needed for QI. While she saw herself as ‘running’ the projects, she needed the ward manager to *lead* the team and manage ward staff (‘She knows the patients better than me. She knows what the ward needs better than me’) and the patient experience officer to provide project support. Here, social capital benefited the collective, but was also a mechanism for accruing further *individual* capital, leading to the promotion of the patient experience officer in question. The social capital accrued where alliances were made between front-line staff and patient experience offices proved salient to the ease with which teams developed and implemented their improvement plans (see table 3).

**Table 3:** Types, definitions and illustrations of capital

Type and definition of capital	Illustration: positive capital	Illustration: negative capital
Economic: Assets and resources such as infrastructure (including office space), equipment and materials, funding, staff capacity and time.	‘Without working with the patient experience department, we wouldn’t have done half the stuff. I mean it's been the fact that we've all worked together that we've got stuff done. Because I haven’t got the time to do it’. (Interview, ward manager) ‘(Name)’d been battling for a year for four new cupboards so the nurses could do the drug round more efficiently…She felt the new Chief Exec had understood something had to be done. As I left the ward, the estates people were there putting in the new cupboards’. (Fieldnote)	‘I: Do you think it's something that can be done within working hours? R: Not on [our unit]. I think if you're on a ward and maybe you're a Band Six and you spend some time in an office, it might be more achievable because you're sat at your desk; you're less likely to be interrupted, and…you don’t have allocated patients…whereas it would be very hard for me to get an hour where I can just be like, 'Right, I'm doing feedback now’ ‘. (Interview, nurse) ‘I ask if they’ve had a chance to sit together since the last learning community event? They haven’t. She thinks they will have to come in outside of work time to meet about the project…because it’s so busy on the shop floor’. (Fieldnote, conversation with nurse)
Social: Access to networks and alliances across different clinical and non-clinical departments	‘We’ve got a good relationship with clinical areas 'cos we help them out with reporting and things. So listening to some of the other Trusts…it almost felt like they, they weren’t, they’re not like scared of the patient experience office, but…it felt like there was no relationship.…Whereas we regularly meet with, communicate with… matrons and sisters. So I don’t think there’s resistance to our office’. (Interview, patient experience project manager) ‘There is quite a lot of laughter at these meetings. The team get on well, they are all allowed to talk and contribute, everyone listens and no-one dominates it. I don’t know whether this is representative of the dynamic on the ward usually or whether these project meetings create a unique space where they feel free to relax’. (Fieldnote)	‘What I have found about certain colleagues on that team is they’re very insular. They like to keep their little achievements to themselves and they don’t like anybody else taking credit for them… I don’t think they thought I was of any relevance from the patient experience side of things. I don’t think they thought I could bring anything to it because I’m not ward-based’. (Interview, patient experience officer) ‘There’s a lot of talk about the head of patient experience and other senior managers, and how they do not communicate with the frontline staff. They’re always on Twitter, but never on the frontline…[Nurse] says it’s notable that it’s only the managers who have time to tweet – junior frontline staff wouldn’t have time during their shift to go on social media, plus they would be berated for it if they did’. (Fieldnote, meeting with frontline team)
Symbolic: Reputation and status within a given setting, influencing ability to recruit, delegate and move agendas forward	‘For a lot of the discharge stuff you would be completely reliant on a doctor engaging with his colleagues and doing the legwork and gathering data…I don’t think it was their expertise that made the difference. I think it was just having them…and the role that they’re in was necessary for the project’. (Interview, senior sister) ‘I: How did they [ward team] benefit? R: Well, they got a big pat on the back, and their picture in the staff magazine. They got the knowledge that they'd done something to improve the safety of their service, and that that was then going on…the intranet, so that people would be contacting them saying 'we've got a similar issue, could we come and have a chat'. (Interview, director of patient experience)	‘We went to this awards ceremony and…this other person said, ‘I’m up for an award because I’ve done x, y and z. I’ve got 30 000 Twitter followers’, all of that jazz… And my colleague said, ‘Oh, well, (name) does a lot of work about dementia’. So we started talking and, yeah, she had a good go at (me)…She just says, kind of in the sense of, I think her words were, ‘You’ve got a lot to learn’… Sometimes it’s a little bit frustrating…I call them 'mood hoovers'. When they suck all the goodness…and life out of you. They’re mood hoovers’. (Interview, activities coordinator) ‘It wasn’t until I went into uniform that people actually started to talk to me…to take me seriously. Because if you’re a person that’s not in uniform and you’re going on the ward…you’re asking them to do something that they see as increasing their job load or whatever, ‘you can’t possibly understand because, you know, you don’t wear a uniform’. (Interview, patient experience officer)
Cultural: Knowledge/expertise in particular forms of practice, for example, medical conditions, administration, patient experience	‘(There's) a huge whiteboard on one wall of her office, completely filled with a brainstorming session on how to take forward quality improvement. It’s a great visual illustration of the energy and activity she puts into patient experience. She refers to it several times in our interview, and it’s clear this is going to be the basis of her masterplan’. (Fieldnote, interview with director of nursing) ‘I:(H)ow were you able to produce [a report to] such a high standard? R: Just over time I guess. Just working in the Trust and working in patient experience, there’s a lot of data sort of working. I’m quite good with Excel. I did a course in it back in high school, a diploma in digital applications…So I’ve got quite a bit of knowledge…It was me that did it. You know what I mean? So it was me that brought it to the table’. (Interview, junior patient experience officer)	‘I'm not sure that the team ever really got to grips with what they were going to do…It always felt like they were doing a bit of pinching with pride from other people. And trying to base what they were going to do on what patients had said, but never really getting there…I think it was ill-thought through, erratic and inconsistent’. (Interview, team member, anonymised) ‘I: Do you feel able to ask someone like [head of patient experience] for data if there was something you wanted to know about? R: I think we might be able to, but having not done it…they probably would question us because we're not management I think. You know, having a healthcare assistant go knocking on the door, 'Can I have information please,' then they’ll probably be like, 'Oh, what do you want information for?’ ‘ (Interview, healthcare assistant)

Another patient experience officer reflected on how positive networking between the patient experience office and clinical ward staff benefited them, compared with other sites involved in the study observed at learning community events. ‘It almost felt like they were […] not like scared of the patient experience office but […] it felt like there was no relationship.’ The word ‘scared’ perhaps reflects a perception of the patient experience team’s role as primarily performance management and inspection rather than support.

This illustrates the negative social capital observed in other sites, where ward teams appeared to have resisted forming significant social alliances, which would have benefitted the team and its improvement projects. By the same token, the loss of significant team members throughout the lifetime of the project also resulted in negative social capital and, in some cases, reduced access to important networks.

### Status, power and recognition—who has symbolic capital?

Symbolic capital relates to an individual’s reputation, prestige, honour and status within a particular social setting. Symbolic capital could be mobilised by teams by, for example, including doctors in their QI projects. The symbolic capital associated with medical prestige was actively sought by one team, which implemented an ambitious redesign of their discharge process. The senior sister reflected that it was not doctors’ medical knowledge per se that was significant, but their role in leveraging wider support (see table 3). In a second example, including a medical registrar as a core team member provided impetus to the project. The registrar’s ability to recruit and delegate project-related activity as part of a ‘junior doctor project’ was directly attributable to the symbolic and social capital associated with that clinical role.

A lack of symbolic capital was evident in the way some staff spoke of a lack of ‘gravitas’ associated with lower NHS staff grade levels. One healthcare assistant commented, for example, that, ‘in general people tend to listen more to a higher banding… than a lower banding’. This was evident across clinical and non-clinical staff, with one patient experience officer suggesting the ward team may have excluded her from project meetings and activities because she did not have sufficient prestige, kudos or clinical standing (despite having a professional background in nursing).

Symbolic capital may nonetheless reside in less ‘powerful’ team members, which in this study included a ward clerk and an activities coordinator. In two of the teams, their status derived from acknowledgement by other team members of their close first-hand knowledge of patient experience on their respective wards (cultural capital) and their ability to channel this to the wider team. However, such symbolic capital could be fragile. The activities coordinator had developed an innovative visual method for reporting and promoting the progress of the team’s project, but explained how he was humiliated at an awards ceremony by a more senior and prestigious clinician from another organisation (see table 3). This criticism was so shaming and demotivating that he ceased all further work on the method. He commented that his staff grade ‘is quite low. And I have to face quite a lot of adversity. And I’ve been really tired.’ The fact that teamwork on the project in this site was strong and diverse (as noted by the ethnographer during year-long observation of QI planning meetings) meant that good progress with QI was maintained despite this incident.

### Forms of knowledge and expertise—contributing cultural capital

Cultural capital is typically manifest in demonstrations of expertise or knowledge. Within the study, this expertise and knowledge sometimes related to clinical/medical knowledge and sometimes to non-clinical expertise in QI methods. For example, a clinical ward manager highlighted the value of the patient experience and QI expertise brought to the ward by the director of patient experience, who had shared her expertise in Experience Based Co-Design with the front-line team before leaving them to run the project. She reflected that collaborative working between the patient experience office and front-line staff across the hospital had led to the production of meaningful knowledge (cultural capital), but also social capital in the form of a network of relationships.

Two of the ward teams involved in the study chose to limit themselves almost entirely to unidisciplinary teamworking, which restricted the type and range of cultural capital available to them when compared with more diverse team membership. It can be argued that a well-functioning unidisciplinary team could form a trusting bond and work more effectively as a result. In one of these two wards, the team was observed to be extremely close-knit and mutually supportive; they were pleased to implement one project (a welcome pack for new patients) which they felt made a real difference. At the same time, they did not draw in other resources and organisational support which could have helped sustain their project longer term and bolster a wider range of actions. Against a background of hostile relations with senior managers, the team trusted each other but nurtured a narrative of embattled suspicion towards others.

This constraint became most evident in the second team, who collectively appeared to have limited time/ability in the skills and knowledge associated with QI methods and struggled with interpreting and using any patient experience data. They, too, focused on one improvement project, the idea for which was borrowed from another site. When asked how they would summarise the type of data the ward team decided to work with, one team member replied, ‘I think it was ill-thought through, erratic and inconsistent’ (see also table 3).

As these examples illustrate, the decision whether or not to include staff with specific skills in QI and patient experience seemed particularly significant in terms of maximising the cultural capital available to teams.

## Discussion

The formation of teams of people from different disciplines and levels of seniority in our study established a network of individuals who brought various aspects of the four forms of capital, generating a form of new, collective ‘team capital’. This enabled them to engage more confidently with analysing and using different types of patient experience data and QI methods (skills which are not commonly taught).^21^ It gave them new insights, and helped them overcome organisational challenges more effectively than teams less able to generate team capital.

Various studies have attempted to explain the characteristics that produce an effective team of health professionals, many of these informed by the science of teamwork and drawing on frameworks from psychology.^3 7–9^ However, the definition of an ‘effective team’ or ‘effective teamwork’ remains contested by academics, policymakers and practitioners.^6^ In a recent paper, Dixon and Wellsteed^22^ evaluated a structured team-based learning approach to QI in two teams: one consisting of nurses and the other of doctors, nurses, therapists, mental health support workers and administrators. Although the latter team was observed to make greater gains in team performance at the end of the intervention, the reasons for this were not explored. The study illustrates the conclusion of a recent scoping review of team dynamics in QI teams which found ‘consensus…that QI teams should be composed of multiple professions; however, the reasons why this kind of composition was necessary were often left unstated’.^11^ The authors conclude that more research is needed into how or why such team diversity impacts the success of QI work.

What this paper adds is the identification of ‘capital’ as a new lens through which to address this question. We suggest that it is a contributory mechanism that can facilitate—or destabilise—the efforts of QI teamwork. It can help us explain the outcomes in each site: what teams were able to achieve, and where they came up against constraints or lack of power. Considering QI team formation through the lens of team capital challenges normal hierarchy, and argues for working relationships that cut across established institutionalised norms and practices. This echoes a finding from Reed *et al*’s ethnography of improvement projects that successful teams ‘tended to be less hierarchical, where the views of all team members were listened to and valued and people were empowered to explore and solve problems’.^21^ Assembling such diverse teams is of course not without risk. It runs counter to evidence from the wider teamwork literature that—while good teamwork is associated with better outcomes for patients—hierarchy and power dynamics between health professions can be a major barrier to true interdisciplinary collaboration.^2 4 6^

## Conclusion

Clearly, team composition is only one factor in successful QI. As Reed *et al* argue, interventions cannot be seen in isolation from a raft of contextual processes and practices.^21^ However, careful identification, engagement and empowerment of appropriate people are a key part of the solution. In practice, we suggest the formation of QI teams could be strengthened by a focus on capital—looking not just at including a range of different people, but mapping more explicitly what kinds of capital they can contribute to institutional teamwork. As part of this, it is important to recognise the value of involving staff such as healthcare assistants, cleaners or porters—people without much formal power in the organisation but rich in insights into the reality of patient experience. Given previous research findings about the challenges of interdisciplinary teamwork in healthcare, we urge caution in not pursuing multidisciplinarity for multidisciplinarity’s sake, but rather focusing on the different forms of capital that different team members can contribute. Further research is needed to test this. We note also that in this project patients and family members were, ironically, conspicuous by their absence in the front-line teams the sites chose to assemble. This may be a missed opportunity to enrich further the range of capital available to teams.
